# Associations of Community Material Neighborhood Deprivation With the Diagnosis of Asthma Among Infants With Bronchopulmonary Dysplasia (BPD)

**DOI:** 10.1002/ppul.71462

**Published:** 2026-01-12

**Authors:** Jonathan J. Szeto, Kathryn Boom, Joshua K. Radack, Sara B. DeMauro, Chén C. Kenyon, Nicolas P. Novick‐Goldstein, Kristan A. Scott, Daria C. Murosko, Kathleen A. Gibbs, Scott A. Lorch, Paul E. Moore, Heather H. Burris, Timothy D. Nelin

**Affiliations:** ^1^ University of Pennsylvania, Perelman School of Medicine Philadelphia PA USA; ^2^ Department of Pediatrics University of Pennsylvania Philadelphia PA USA; ^3^ Department of Pediatrics Vanderbilt University School of Medicine Nashville TN USA

## Abstract

**Objective:**

To quantify associations of the community‐level material deprivation index (CMDI) with asthma diagnosis by age 5 years among preterm infants with bronchopulmonary dysplasia (BPD).

**Methods:**

We conducted a retrospective cohort study of preterm infants with BPD, born between 2010 and 2019, discharged from a single hospital system to a home address in the Philadelphia metropolitan area, with documented follow‐up in the Children's Hospital of Philadelphia Care Network through 5 years of age. Patient charts were reviewed for asthma diagnoses, identified by ICD‐10 codes. We geocoded each patient's address at time of neonatal intensive care unit (NICU) discharge to assign census tract CMDI values (range 0 to 1). Multivariable logistic regression models quantified associations of CMDI with asthma diagnosis by age 5 adjusting for patient‐level factors.

**Results:**

Of the 337 preterm infants with BPD and 5‐year follow‐up within the CHOP Care Network, 169 (50%) were diagnosed with asthma by age 5. CMDI was higher among infants diagnosed with asthma compared to those without asthma (0.43 vs 0.38, *p* = 0.002). Per standard deviation increment of CMDI, infants had 34% and 32% higher odds of asthma diagnosis in unadjusted (OR 1.34, 95% CI: 1.11, 1.62) and adjusted (aOR 1.32, 95%CI: 1.05–1.65) models, respectively.

**Conclusions:**

Among an urban population of former preterm infants with BPD, high rates of asthma by school age were noted and higher neighborhood deprivation was associated with asthma diagnosis by age 5 years.

## 1 Introduction

Bronchopulmonary dysplasia (BPD) is the most common chronic respiratory morbidity of preterm birth [1]. Infants born < 32 weeks gestational age often have underdeveloped lungs requiring supplemental oxygen and ventilation support. These interventions can lead to pulmonary inflammation, scarring, and, ultimately, BPD. The lungs of infants with BPD can show pathological evidence of necrotizing bronchiolitis, pulmonary hypertension, inflammatory response, fibrosis, and both alveolar overinflation and atelectasis [2]. Structural alterations in lung parenchyma and pulmonary vasculature can lead to enduring impairments in gas exchange and cardiopulmonary function [3, 4, 5]. As such, infants with BPD interact frequently with the healthcare system after the initial stay in the neonatal intensive care unit (NICU), posing significant burdens on families' quality of life and finances [6, 7].

Asthma, characterized by airway inflammation and constriction, is the most common chronic lung disease among children in the United States [8]. Preterm infants with BPD have three times the prevalence of childhood asthma compared to the general population [9, 10]. Social determinants of health are significantly associated with childhood asthma, with children residing in areas of greater material deprivation having increased asthma diagnosis risk, asthma medication utilization, and healthcare utilization for asthma exacerbations [11, 12, 13, 14, 15].

Given that maternal neighborhood deprivation is associated with the risk of preterm birth, infants with BPD are disproportionately discharged from the NICU to socially vulnerable neighborhoods [16]. As such, there is a heightened concern for how community‐level factors may influence health outcomes among this already vulnerable population of infants. Prior studies by our group and others have demonstrated that neighborhood‐level factors, both social and environmental, are associated with acute adverse health outcomes among preterm infants with BPD [17, 18, 19, 20, 21]. However, a knowledge gap exists in understanding how these same neighborhood‐level factors are associated with the development of chronic pediatric conditions commonly associated with BPD. Specifically, whether neighborhood socioeconomic factors at time of NICU discharge are associated with an increased risk of asthma among infants with BPD is unknown. As such, we sought to quantify the association of community‐level material deprivation with the diagnosis of asthma by 5 years of age among infants with BPD.

## 2 Methods

### 2.1 Study Population

We conducted a retrospective cohort study of infants with BPD, born between 2010 and 2019, discharged from a single hospital system to a home address in the metropolitan Philadelphia area, with documented receipt of care in the Children's Hospital of Philadelphia (CHOP) Care Network through the age of five. Infants were included if they received any type of care including primary care, subspecialty care, acute care (e.g., urgent care or emergency department), or inpatient care at any CHOP‐affiliated site through at least 5 years of age. To ensure longitudinal follow‐up, infants were excluded if they did not have documented care encounters extending to or beyond age five; for example, infants with only a single follow‐up visit at age two and no subsequent visits were excluded. Infants missing documentation of home address at time of hospital discharge and those discharged to an address outside of the Philadelphia metropolitan region were excluded.

Patient data were abstracted from the CHOP electronic health record. CHOP serves as the inpatient and post‐discharge medical home for most infants with high‐grade BPD in the region. Infants were included if they were born at less than 32 weeks' gestation between January 1st, 2010 and December 31st, 2019, survived until hospital discharge, and diagnosed with BPD using the 2019 Neonatal Research Network BPD definition. BPD severity was assigned based on respiratory support at 36 weeks' postmenstrual age (PMA), or at the time of CHOP NICU admission if transferred after 36 weeks' PMA, and categorized as grade 1 (nasal cannula ≤ 2 L/min), grade 2 (noninvasive support or nasal cannula > 2 L/min), or grade 3 (invasive ventilation) [23]. Infants capable of breathing room air without supplemental respiratory support at 36 weeks' PMA (regardless of prior respiratory support usage) are not diagnosed with BPD and thus were excluded from this study. A flowchart of cohort inclusion is presented in Supplemental Figure 1 and characteristics of excluded infants are provided in Supplemental Table 1.

### 2.2 Exposures and Outcomes

The Community‐Material Deprivation Index (CMDI) quantifies deprivation for each census tract in the United States based on a principal component analysis of six different 2018 American Community Survey measures [24, 25]. The CMDI was used in this analysis as it has been validated in a pediatric population and previously used to measure the association of community material deprivation with health care utilization among infants [24]. CMDI measures include fraction of the census tract population with assisted income, fraction of the census tract population with a high school education, fraction of the census tract population with no health insurance, fraction of the census tract population in poverty, fraction of housing in the census tract that is vacant, and the median income. The CMDI is constructed using principal component analysis (PCA) of these six standardized variables from the American Community Survey. Each variable is normalized (typically via z‐scores) and directionally aligned so that higher values consistently reflect greater deprivation. The first principal component—which captures the most shared variance across these domains—is then rescaled to range from 0 to 1 across all U.S. census tracts, with higher scores indicating greater material deprivation [24]. Each infant's residential address at time of NICU discharge was geocoded using ArcMAP Version 10.8 and ArcGIS Street Map Premium North America 2021.1 address locator, using a minimum match score of 75 and assigning a census tract based on the 2010 U.S. Census boundaries. We used each patient's geocoded address at time of NICU discharge to assign census tract CMDI values. This timepoint was selected to evaluate whether early‐life community‐level exposures—proximal to the neonatal period—may contribute to asthma risk, capturing a critical developmental window.

The primary study outcome was the diagnosis of asthma by 5 years of age. We identified diagnoses of asthma through a two‐step process. Asthma diagnoses were first defined as at least one documented diagnosis using International Classification of Diseases, Tenth Revision (ICD‐9/10), asthma codes (493.x/J45.x, available in Supplemental Table 2). To improve diagnostic accuracy, we then conducted a manual chart review within the Epic electronic health system. We used the search bar function to query the term “asthma” across clinical documentation, including provider notes, associated diagnoses for medication prescriptions, and clinical encounters. Manual chart review was performed by two independent reviewers (JJZ and TDN), with discrepancies resolved by consensus. Asthma diagnosis was assigned to infants if they met either ICD‐9/10 or chart review criteria. All infants who were assessed to have asthma by ICD code also had confirmatory evidence on chart review. The cutoff age of five was chosen because most children will have their first asthma symptoms by this point, with past epidemiological studies indicating asthma incidence rates peak in the 0–4 year age group, then decline with age [26, 27].

### 2.3 Statistical Analysis

We performed descriptive bivariable analyses of associations of infant characteristics with the development of asthma by age 5. Continuous variables were analyzed with Student's two‐sided *t* tests and Wilcoxon rank sum tests. Categorical variables were analyzed with Chi‐square tests.

Multivariable logistic regression models quantified associations of one standard deviation (SD)‐unit increment higher CMDI with the diagnosis of asthma by age 5 with robust variance estimates, adjusting for the following patient‐level factors: gestational age; sex; birth weight; BPD grade; discharge age; year of discharge; discharge inhaled medication; discharge respiratory support; and insurance type [9, 10, 14, 18, 28, 29, 30]. As our objective was to assess the association of neighborhood deprivation with asthma diagnosis among infants with BPD, not to examine racial disparities in asthma diagnosis or evaluate whether exposure to differing levels of neighborhood deprivation mediates racial disparities in respiratory outcomes among preterm infants with BPD, we elected not to adjust for race and ethnicity in the primary analysis as race and ethnicity are non‐modifiable characteristics and do not represent biological confounders, instead serving as proxies for exposure to inequitable structural conditions [31, 32]. While CMDI values range from 0 to 1, individual unit changes (e.g., 0.05 or 0.1) can be difficult to interpret intuitively, given the index's composite and standardized nature. To aid interpretability and comparability, we modeled CMDI as a continuous variable per standard deviation (SD) increment. This approach allows us to summarize the association of a shift in neighborhood deprivation – capturing cumulative socioeconomic disadvantage across six domains – with increased odds of asthma diagnosis by 5 years of age, rather than focusing on absolute value changes, which may not translate directly to individual family experiences.

We conducted a secondary analysis restricted to infants with BPD discharged NICU without any inhaled respiratory medications or supplemental respiratory support, since the presence of these medications may prompt physicians and coders to use asthma ICD‐10 codes in the absence of asthma symptoms. We then conducted multiple *post hoc* sensitivity analyses to assess the robustness of our findings to restrict the study population (1) to infants with a general pediatrician in the CHOP Primary Care Network to assess the definition and validity of the primary outcome, (2) to infants with a 100% geocoding match score to address geocoding precision and exposure misclassification, (3) to infants discharged 2013–2019 to address temporal alignment of CMDI values, (4) to infants who did not move census tracts in the first year after NICU discharge to address early‐life residential mobility.

This research was approved by the Institutional Review Board (IRB) of CHOP (IRB 20‐018358). Given the retrospective nature of the study, the IRB did not require informed consent. This study is reported in accordance with the STROBE (Strengthening the Reporting of Observational Studies in Epidemiology) statement, using the checklist for cohort studies to guide transparent reporting. All analyses were performed with Stata (Stata 18, College Station, TX, USA). Code available at https://github.com/tnelin/CMDI-and-Asthma.git.

## 3 Results

**Figure 1:**
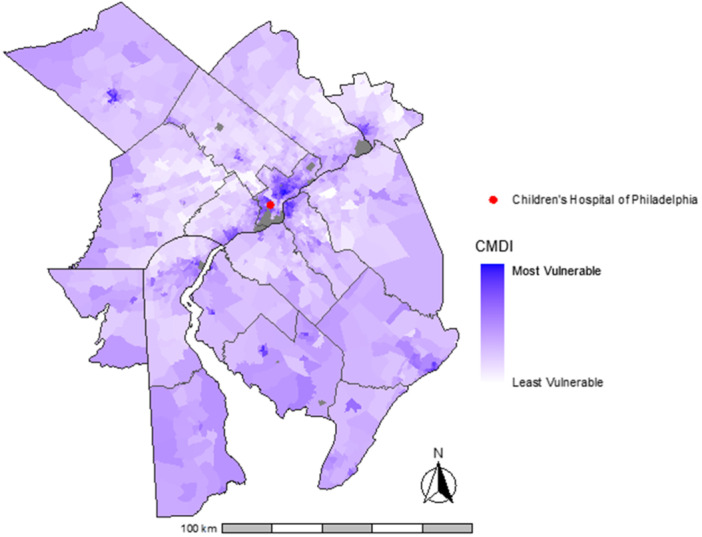
Cohort community material deprivation index by census tract across the philadelphia metropolitan area, Brokamp et al. 2019 [22]. Six variables from the American Community Survey are used to create the Community Material Deprivation Index: fraction of the population with income in the past 12 months below the poverty level, median household income in the past 12 months, fraction of the population 25 and older with attainment of at least a high school degree, fraction of the population with no health insurance, fraction of households receiving public assistance income or food stamps or SNAP in the past 12 months, and fraction of houses that are vacant. The nationwide mean for the 2018 deprivation index was 0.35. The cohort mean for the 2018 deprivation index in metropolitan Philadelphia was 0.41. [Color figure can be viewed at wileyonlinelibrary.com]

Of the 337 infants with BPD for whom follow‐up data through 5 years were available, 169 (50.1%) were diagnosed with asthma by age 5. Figure 1 displays variation in census tract CMDI across the metropolitan Philadelphia region for the cohort. Table 1 displays cohort characteristics. The mean (SD) CMDI of the cohort was 0.41 (0.16). CMDI was higher among infants diagnosed with asthma (0.43 vs 0.38, *p* = 0.002). Breaking the CMDI down into its six‐component metrics, infants and children diagnosed with asthma by age 5 resided in census tracts with a greater percentage of individuals on assisted income, without health insurance, in poverty, higher percentages of vacant housing, and lower levels of high school education attainment and median income (Table 2). Clinical characteristics of infants without follow‐up through age 5 in the CHOP Care Network are shown in Supplemental Table 1.

**Table 1:** Cohort demographic and clinical characteristics among preterm infants with bronchopulmonary dysplasia, stratified by asthma diagnosis by age 5 years.

	Overall	Asthma diagnosis	No asthma diagnosis	*p* value
	(*n* = 337)	(*n* = 169)	(*n* = 168)	
Infant demographic characteristics				
Male sex, *n* (%)	199 (59.1)	100 (59.2)	99 (58.9)	0.960
Infant race/ethnicity, *n* (%)				0.026
Non‐hispanic black	174 (51.6)	99 (58.6)	75 (44.6)	
Non‐hispanic white	88 (26.1)	35 (20.7)	53 (31.5)	
Other	75 (22.3)	35 (20.7)	40 (23.8)	
Hispanic ethnicity, *n* (%)	35.0 (10.4)	21.0 (12.4)	14.0 (8.3)	0.220
Public insurance, *n* (%)	208 (61.7)	112 (66.3)	96.0 (57.1)	0.085
Infant clinical characteristics—NICU admission				
Gestational age, weeks, median [IQR]	27 [24–28]	26 [25–27]	27 [25–29]	0.004
Birth weight, grams, median [IQR]	771 [630–1015]	737 [609–930]	820 [643–1073]	0.034
BPD severity				0.570
Grade 1	120 (35.6)	56.0 (33.1)	64.0 (38.1)	
Grade 2	133 (39.5)	71.0 (42.0)	62.0 (36.9)	
Grade 3	84 (24.9)	42.0 (24.9)	42.0 (25.0)	
Infant clinical characteristics—NICU discharge				
Discharge age, days, median [IQR]	132 [95–198]	138 [98–207]	129 [92–191]	0.550
Discharge Inhaled Medications, *n* (%)				0.008
Albuterol	58 (17.2)	27 (16.0)	31 (18.5)	
Albuterol + inhaled corticosteroids	37 (11.0)	26 (15.4)	11 (6.6)	
None	242 (71.8)	116 (68.6)	126 (75.0)	
Discharge support, *n* (%)				0.81
Tracheostomy	46 (13.6)	23 (13.6)	23 (13.7)	
Supplemental oxygen	40 (11.9)	22 (13.0)	18 (10.7)	
None	251 (74.5)	124 (73.4)	127 (75.6)	

Abbreviations: BPD, Bronchopulmonary dysplasia; CMDI, community‐level material deprivation; IQR, interquartile range; SD, standard deviation.

**Table 2:** Cohort community‐level deprivation index among preterm infants with bronchopulmonary dysplasia, stratified by asthma diagnosis by age 5 years.

	Overall	Asthma diagnosis	No asthma diagnosis	*p* value
	(*n* = 337)	(*n* = 169)	(*n* = 168)	
Community‐level material deprivation index, mean (SD)	0.40 (0.16)	0.43 (0.16)	0.38 (0.16)	0.002
Fraction with assisted income	23.3 (0.2)	26.1 (18.1)	20.5 (16.8)	0.004
Fraction with high school degree	85.7 (0.1)	84.4 (10.0)	87.9 (8.8)	0.006
Fraction without health insurance	8.7 (0.05)	9.5 (5.6)	7.8 (5.1)	0.004
Fraction population in poverty	20.0 (0.2)	22.0 (14.8)	18.1 (14.4)	0.015
Fraction of vacant houses	12.0 (0.1)	12.6 (7.2)	11.4 (8.2)	0.16
Household income, $, median [IQR]	50,298 [32,027, 77,143]	42,500 [30,328, 74,352]	57,250 [34,549, 81,837]	0.004

### 3.1 CMDI and Asthma Diagnosis

**Figure 2:**
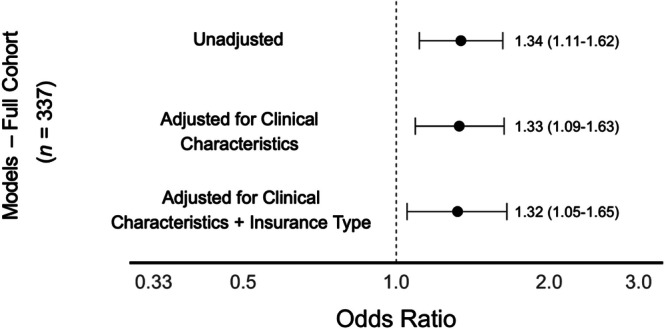
Associations of community‐level material deprivation index (CMDI) with asthma diagnosis among all included preterm infants with bronchopulmonary dysplasia (*n* = 337). Odds ratios presented per standard deviation increment of CMDI. Clinical characteristics include gestational age, sex, birth weight, bronchopulmonary dysplasia severity, discharge age, and year of discharge.

**Figure 3:**
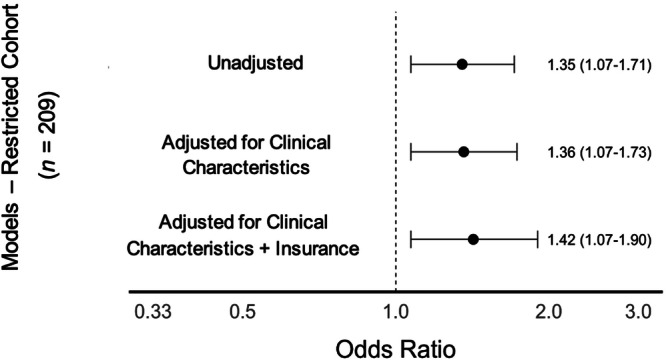
Associations of community‐level material deprivation index (CMDI) with asthma diagnosis among the restricted cohort of preterm infants with bronchopulmonary dysplasia (*n* = 209), discharged from the NICU without mechanical respiratory support or inhaled medications. Odds ratios presented per standard deviation increment of CMDI. Clinical characteristics include gestational age, sex, birth weight, bronchopulmonary dysplasia severity, discharge age, and year of discharge.

In unadjusted analysis, infants had 34% higher odds of asthma diagnosis by age 5 (OR: 1.34, CI: 1.11–1.62) per standard deviation increment of census tract CMDI. After multivariable adjustment for infant clinical characteristics and insurance type, this association persisted (aOR 1.32, CI: 1.05–1.65) (Figure 2). Secondary analysis of the 214 infants with BPD discharged from the NICU without inhaled respiratory medications or supplemental mechanical respiratory support had similar associations in unadjusted (OR: 1.37, CI: 1.07–1.71) and adjusted models (aOR: 1.42, 95% CI: 1.07–1.90) (Figure 3).

In *post‐hoc* sensitivity analyses that restricted to infants (1) with a general pediatrician in the CHOP Primary Care Network, (2) with a 100% geocoding match score, (3) discharged 2013‐2019, and (4) who did not move census tracts in the first year after NICU discharge, the association of CMDI with asthma diagnosis was similar in direction and magnitude, with adjusted odds ratios ranging from 1.26 to 1.38 per standard deviation increase (Supplemental Tables 3–6).

## 4 Discussion

In this retrospective cohort study of preterm infants with BPD, infants discharged to addresses in census tracts with higher community‐level material deprivation had increased odds of asthma diagnosis by 5 years of age. This association persisted with adjustment for infant clinical characteristics and insurance type. The association also persisted when restricting to infants discharged from the NICU without any respiratory medication or support and was similar in direction and magnitude in *post‐hoc* sensitivity analyses to demonstrate the robustness of our findings.

CMDI reflects neighborhood‐level material deprivation and is derived at the census tract level, capturing structural disadvantage across domains such as income, education, insurance coverage, and housing [24]. As the index is standardized and composite, small absolute differences (e.g., 0.05 or 0.1) may not correspond to meaningful changes at the individual level. The unadjusted difference in CMDI between infants with and without asthma in our cohort (0.43 vs 0.38) appears modest; however, the index was modeled as a continuous exposure scaled per one standard deviation. Modeling per standard deviation increment allows for a more interpretable assessment of the association of neighborhood deprivation with asthma. In our cohort the CMDI standard deviation was 0.16, which is similar to the national CMDI distribution [24]. A one‐standard‐deviation increment reflects a more meaningful shift along the deprivation gradient in Philadelphia, corresponding to differences in poverty concentration, educational attainment, housing vacancy, and other material resources at the census‐tract level. Per standard deviation increment of CMDI, the adjusted odds ratio was 1.32 (95% CI: 1.05–1.65). These findings reflect population‐level associations, highlighting how structural inequities may shape chronic respiratory outcomes among former preterm infants with BPD. At the same time and importantly for the context of improving care for infants with BPD, physiologic factors such as degree of prematurity, birthweight, dependency on supplemental oxygen or a tracheostomy, likely remain the primary contributors to respiratory vulnerability among infants with BPD, and neighborhood deprivation should be viewed as a modifiable and additional but likely smaller contextual factor.

Our findings are consistent with evidence that social vulnerability is a risk factor for development of asthma in children [11, 12, 13, 14, 15]. Specifically, Lucas et al. investigated associations of neighborhood deprivation with albuterol prescriptions in a cohort of 34,266 children and found that children from the most deprived neighborhoods had increased rates of albuterol prescription compared to infants from the least deprived neighborhoods (rate ratio = 1.22, 95% CI: 1.13, 1.32) [12]. More recently, Miller et al. utilized data from the Environmental Influences on Child Health Outcomes program to investigate the association of child opportunity index with incidence rates for asthma with recurrent exacerbation among 15,877 children and found that rates of asthma with recurrent exacerbation were higher among children born in under‐resourced communities (Incidence Ratio = 10.98; 95% CI: 9.71, 12.25 for very low child opportunity index neighborhoods) [15]. Our analysis adds to the literature demonstrating that the social vulnerability of a neighborhood is an additional factor associated with increased risk of asthma among already vulnerable infants.

An alternative explanation for our findings is that community‐level deprivation may influence patterns of healthcare utilization and diagnostic labeling rather than underlying pathophysiologic asthma. Infants living in more deprived neighborhoods often experience higher burdens of unmet social needs, challenges in accessing primary care, and greater reliance on acute care settings, where diagnostic practices and thresholds may vary [33, 34, 35]. These factors could contribute to increased opportunities for receiving an asthma diagnosis independent of true differences in airway disease. While our sensitivity analysis restricting the cohort to infants discharged without inhaled medications or supplemental respiratory support reduces the likelihood that diagnostic labeling alone accounts for the association, this possibility cannot be fully excluded. Neighborhood deprivation may therefore reflect both contextual exposures that influence respiratory vulnerability and structural factors that shape when and how respiratory symptoms are evaluated and coded within the healthcare system.

Our findings are biologically plausible. Infants residing in houses of poorer quality with more adverse neighborhood exposures are exposed to more environmental triggers, which may provoke asthma symptoms such as cough, wheeze, and difficulty breathing [36, 37]. Though housing quality is not an exact metric within the CMDI, nearby vacant housing and area‐level poverty may act as a suitable surrogate. Given that material deprivation has already been established as a risk factor for infants developing asthma [11, 12, 13, 14, 15], many of the same principles and practices that have been employed to reduce these social risks in term infants with asthma can likely also be applied to preterm infants with BPD [35]. While there are no existing interventions to prevent incident asthma, multidisciplinary asthma clinics with robust social work teams can decrease healthcare utilization among their patients diagnosed with asthma [13]. However, it is important to note that while neighborhood indices may be poor proxies for household‐level screening, a recent study by Luke et al. found that Child‐Opportunity Index, another neighborhood index, had a low positive predictive value, but high negative predictive value for identifying individual level social needs among children admitted to a quaternary children's hospital [34]. Further, Beck et al. highlight reductions in hospitalizations among children referred to a primary‐care based medical‐legal partnership and hypothesized that the reductions were secondary to the ability of the advocates and resources at the medical‐legal partnership to address acute needs and confront the root causes of adverse health outcomes [38]. Integrating a similar framework with resources to help families address their unmet social needs into BPD‐specific follow‐up clinics and integrating BPD as a diagnosis associated with high risk of unmet social needs in a hospital system electronic health record represent two strategies that could be employed.

A key strength of the study was measuring neighborhood deprivation based on the census tract of residence at NICU discharge. This approach strengthened the temporal relationship between early‐life neighborhood exposure and asthma risk, while also raising the important question of whether exposures earlier in the life course—proximal to NICU discharge—may also contribute to the diagnosis of asthma among these high‐risk infants. Additional strengths of this study include its relatively large sample size spanning a 10‐year period and the use of standardized, evidence‐based definitions to identify BPD and categorize its severity. To our knowledge, this is the first study to examine the association of CMDI with asthma specifically among infants with BPD. Asthma is a common, long‐term morbidity among these infants and our findings highlight a novel intersection between social determinants of health and chronic respiratory outcomes in this high‐risk population. In addition, there is concern that children with BPD experience earlier onset COPD and adult respiratory disease than their counterparts born at full‐term [39, 40]. It is unknown whether asthma will exacerbate this process so this finding may carry important lifelong and even potentially life limiting implications [40]. These findings open the door to future investigations into how neighborhood deprivation may influence other critical outcomes in infants with BPD, including neurodevelopment, pulmonary hypertension, and nutritional status. Further, our secondary analysis, which restricted the cohort to infants not discharged with supplemental respiratory support or inhaled medications, enhances internal validity by addressing a potential confounder—early airway reactivity—of the pathway from BPD to asthma diagnosis. The consistency of results across multiple post‐hoc sensitivity analyses indicates that the observed CMDI‐asthma association is robust across different analytic specifications to address the potential of misclassification, temporal mismatch, and instability of early‐life residential environments.

This study has several limitations. First, the retrospective design to investigate how neighborhood social vulnerability at time of NICU discharge was associated with early childhood asthma diagnosis limited examination of social circumstances over time. While this was an intentional decision to model the early life exposure before the outcome and results of the *post‐hoc* sensitivity analysis restricted to infants who did not move census tracts within the first year after NICU discharge revealed an association of CMDI with the diagnosis of asthma in the same direction and magnitude, we did not account for residential mobility beyond the first year after NICU discharge. Second, the study population was drawn from a single metropolitan area and limited to infants with follow‐up within one health system which may reduce the generalizability of our findings to other regions or care settings. Third, asthma diagnosis was partially determined based on ICD‐9/10 codes in the medical record and we did not adjudicate who made the diagnosis or whether diagnostic criteria were uniformly applied, however this reflects the real‐world practice of how infants with BPD interact with numerous facets of the healthcare system. ICD‐9/10 codes may lead to outcome misclassification; however, we utilized a two‐step approach including manual chart reviews within the electronic health record to minimize this limitation, and the similar findings in our secondary analysis restricted to infants without inhaled therapies at NICU discharge are reassuring. Fourth, there is potential for ecological fallacy as area level characteristics do not always reflect individual level exposures. Fifth, while American Academy of Pediatrics consensus guidelines discourage routine use of inhaled bronchodilators or corticosteroids in infants with BPD [41], nearly 30% of infants in the cohort were discharged with albuterol or inhaled corticosteroids, which may reflect practice variation and create potential diagnostic reinforcement bias as clinicians may be more likely to diagnose these infants with asthma. Importantly, we adjusted for inhaled medication use at time of discharge, but did not have access to high quality medication usage data after time of NICU discharge to assess how if these medications were discontinued shortly after discharge or continued chronically. Finally, study inclusion required follow‐up within the CHOP Care Network through age five, which may have introduced selection bias by excluding 41 infants without follow‐up data within the CHOP Care Network had higher rates of severe BPD, tracheostomy, and inhaled medication use with lower CMDI. However, since this is a small proportion of infants, there is a low likelihood that this would significantly alter the results of the analysis.

## 5 Conclusion

Among preterm infants with BPD, being discharged to a neighborhood of higher community‐level deprivation was associated with asthma diagnosis by 5 years of age, even after controlling for clinical characteristics and insurance type. Future studies should utilize geographically diverse cohorts to obtain more generalizable results and investigate the associations neighborhood deprivation with the development of other chronic health conditions associated with BPD, such as neurological issues, developmental delays, and feeding issues. In addition, investigation into the specific ways in which neighborhood deprivation leads to unmet social needs can help providers, healthcare systems, and larger scale policies to intervene to improve long‐term health among preterm infants with BPD.

## Author Contributions

Jonathan J. Szeto, Timothy D. Nelin, and Heather H. Burris conceptualized and designed the study, coordinated, supervised, and collected data, carried out analysis, interpret the data and analysis results. Jonathan J. Szeto wrote the initial manuscript draft and reviewed and revised the manuscript throughout the submission process. Timothy D. Nelin and Heather H. Burris reviewed and revised the initial draft and were involved with revisions throughout the submission process. Kathryn Boom, Joshua K. Radack, Sara B. DeMauro, Chén C. Kenyon, Nicolas P. Novick‐Goldstein, Kristan A. Scott, Daria C. Murosko, Kathleen A. Gibbs, Paul E. Moore and Scott A. Lorch helped conceptualize the study, reviewed, and revised the manuscript throughout the submission process.

## Conflicts of Interest

The authors declare no conflicts of interest.

## Supporting information


**Supplemental Figure 1**: Study inclusion flowchart. **Supplemental Table 1:** Characteristics of 41 infants with a discharge address in the metropolitan Philadelphia region without documented follow‐up through age 5 in the CHOP Care Network. **Supplemental Table 2:** Asthma ICD‐9/10 codes used in data query. **Supplemental Table 3:** Association of community‐level material deprivation (CMDI) with asthma diagnosis by age 5 years, restricted to infants with continuous primary care follow‐up in the CHOP Primary Care Network (n = 233). **Supplemental Table 4:** Association of community‐level material deprivation (CMDI) with asthma diagnosis by age 5 years, restricted to infants with 100% geocoding match score (n = 288). **Supplemental Table 5:** Association of community‐level material deprivation (CMDI) with asthma diagnosis by age 5 years, restricted to infants discharged 2013‐2019 (n = 285). **Supplemental Table 6:** Association of community‐level material deprivation (CMDI) with asthma diagnosis by age 5 years, restricted to infants without change in residential census tract in the first year after NICU discharge (n = 223).

## Data Availability

The datasets generated during/or analyzed during the current study and the code used to analyze and manage the data are available from the corresponding author on reasonable request.
