# Supplementary material for: Associations of Community Material Neighborhood Deprivation With the Diagnosis of Asthma Among Infants With Bronchopulmonary Dysplasia (BPD)
Source: Pediatr Pulmonol. 2026 Jan 12;61(1):e71462. doi: 10.1002/ppul.71462 (PMC12794746; doi:10.1002/ppul.71462)
Supplement: Supplementary file 1 — Supplemental Figure 1: Study inclusion flowchart. Supplemental Table 1: Characteristics of 41 infants with a discharge address in the metropolitan Philadelphia region without documented follow‐up through age 5 in the CHOP Care Network. Supplemental Table 2: Asthma ICD‐9/10 codes used in data query. Supplemental Table 3: Association of community‐level material deprivation (CMDI) with asthma diagnosis by age 5 years, restricted to infants with continuous primary care follow‐up in the CHOP Primary Care Network (n = 233). Supplemental Table 4: Association of community‐level material deprivation (CMDI) with asthma diagnosis by age 5 years, restricted to infants with 100% geocoding match score (n = 288). Supplemental Table 5: Association of community‐level material deprivation (CMDI) with asthma diagnosis by age 5 years, restricted to infants discharged 2013‐2019 (n = 285). Supplemental Table 6: Association of community‐level material deprivation (CMDI) with asthma diagnosis by age 5 years, restricted to infants without change in residential census tract in the first year after NICU discharge (n = 223). [file PPUL-61-0-s001.docx]

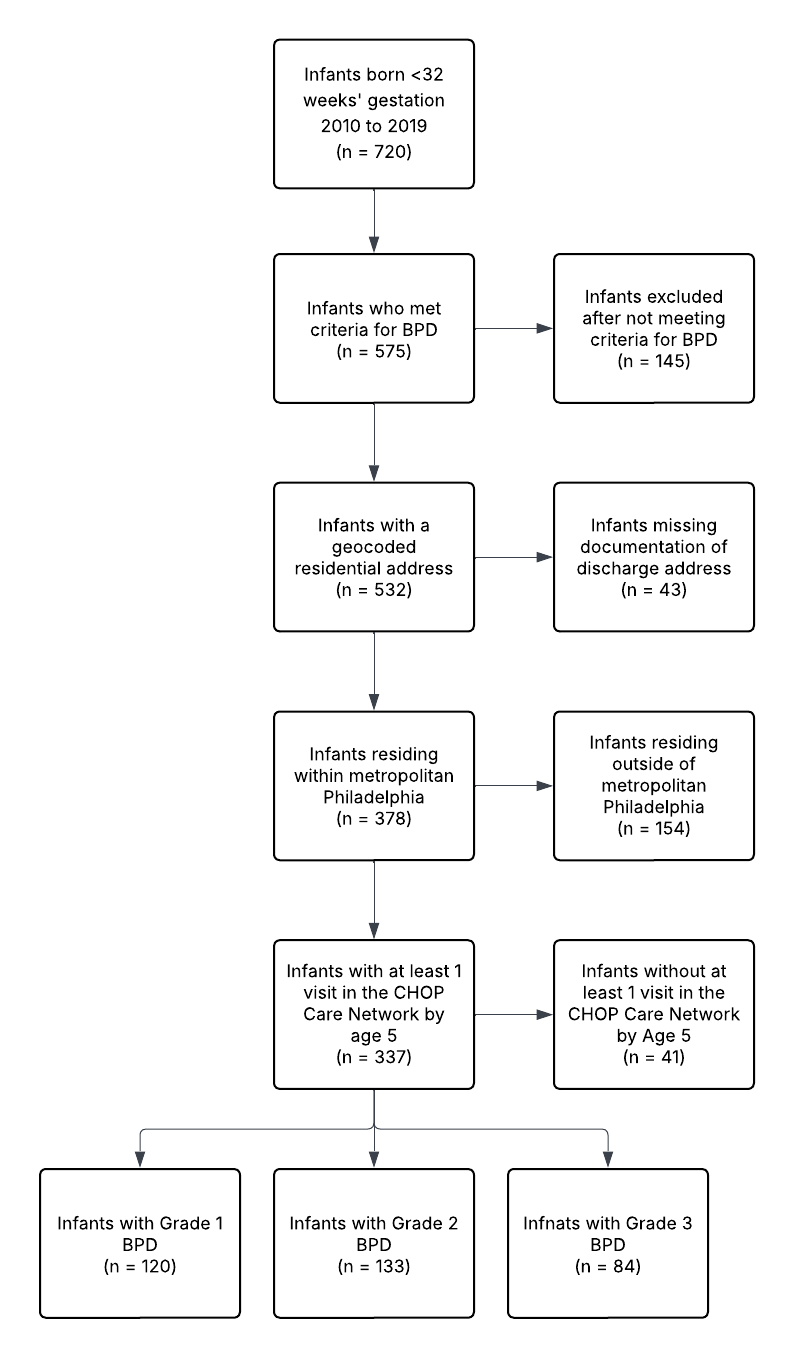


**Supplemental Figure 1**. Study inclusion flowchart.

| **Infant Characteristic** | **Overall (n = 41)** |
| --- | --- |
| Gestational Age, weeks, median [IQR] | 28 [25–32] |
| Birth Weight, grams, median [IQR] | 802 [660–1395] |
| Male sex, n (%) | 21 (51) |
| BPD Grade, n (%) |  |
| Grade 1 | 7 (17) |
| Grade 2 | 20 (49) |
| Grade 3 | 14 (34) |
| Discharge age, days, median [IQR] | 115 [87–192] |
| Discharge Support, n (%) |  |
| Tracheostomy | 7 (17) |
| Supplemental Oxygen | 5 (12) |
| None | 29 (71) |
| Discharge inhaled medications, n (%) |  |
| Albuterol | 15 (37) |
| Albuterol + Inhaled Corticosteroids | 6 (15) |
| None | 20 (48) |
| Public Insurance, n (%) | 27 (66) |
| Community Material Deprivation Index, mean (SD) | 0.33 (0.15) |

**Supplemental Table 1.** Characteristics of 41 infants with a discharge address in the metropolitan Philadelphia region without documented follow-up through age 5 in the CHOP Care Network.

| **ICD-10 Code** | **Description** | **ICD-9 Code** | **Description** |
| --- | --- | --- | --- |
| J45.20 | Mild intermittent asthma, uncomplicated | 493.00 | Extrinsic asthma, unspecified |
| J45.21 | Mild intermittent asthma with (acute) exacerbation | 493.01 | Extrinsic asthma with status asthmaticus |
| J45.22 | Mild intermittent asthma with status asthmaticus | 493.02 | Extrinsic asthma with (acute) exacerbation |
| J45.30 | Mild persistent asthma, uncomplicated | 493.10 | Intrinsic asthma, unspecified |
| J45.31 | Mild persistent asthma with (acute) exacerbation | 493.11 | Intrinsic asthma with status asthmaticus |
| J45.32 | Mild persistent asthma with status asthmaticus | 493.12 | Intrinsic asthma with (acute) exacerbation |
| J45.40 | Moderate persistent asthma, uncomplicated | 493.20 | Chronic obstructive asthma, unspecified |
| J45.41 | Moderate persistent asthma with (acute) exacerbation | 493.21 | Chronic obstructive asthma with status asthmaticus |
| J45.42 | Moderate persistent asthma with status asthmaticus | 493.22 | Chronic obstructive asthma with (acute) exacerbation |
| J45.50 | Severe persistent asthma, uncomplicated | 493.81 | Exercise-induced bronchospasm |
| J45.51 | Severe persistent asthma with (acute) exacerbation | 493.82 | Cough-variant asthma |
| J45.52 | Severe persistent asthma with status asthmaticus | 493.90 | Asthma, unspecified |
| J45.909 | Unspecified asthma, uncomplicated | 493.91 | Asthma, unspecified with status asthmaticus |
| J45.901 | Unspecified asthma with (acute) exacerbation | 493.92 | Asthma, unspecified with (acute) exacerbation |
| J45.902 | Unspecified asthma with status asthmaticus |  |  |

**Supplemental Table 2.** Asthma ICD-9/10 codes used in data query.

**Supplemental Table 3.** Association of community-level material deprivation (CMDI) with asthma diagnosis by age 5 years, restricted to infants with continuous primary care follow-up in the CHOP Primary Care Network (n = 233).

|  | **OR** | **95% CI** | ***p*-value** |
| --- | --- | --- | --- |
| Unadjusted | 1.34 | 1.07-1.67 | 0.012 |
| Adjusted* | 1.26 | 0.93-1.69 | 0.137 |

*Model adjusted for gestational age, birth weight, BPD grade, discharge age (days), discharge inhaled medication category, discharge respiratory support, birth year, sex, and insurance type.

**Supplemental Table 4.** Association of community-level material deprivation (CMDI) with asthma diagnosis by age 5 years, restricted to infants with 100% geocoding match score (n = 288).

|  | **OR** | **95% CI** | ***p*-value** |
| --- | --- | --- | --- |
| Unadjusted | 1.36 | 1.11-1.67 | 0.003 |
| Adjusted* | 1.36 | 1.06-1.75 | 0.017 |

*Model adjusted for gestational age, birth weight, BPD grade, discharge age (days), discharge inhaled medication category, discharge respiratory support, birth year, sex, and insurance type.

**Supplemental Table 5.** Association of community-level material deprivation (CMDI) with asthma diagnosis by age 5 years, restricted to infants discharged 2013-2019 (n = 285).

|  | **OR** | **95% CI** | ***p*-value** |
| --- | --- | --- | --- |
| Unadjusted | 1.33 | 1.08-1.64 | 0.007 |
| Adjusted* | 1.36 | 1.05-1.74 | 0.018 |

*Model adjusted for gestational age, birth weight, BPD grade, discharge age (days), discharge inhaled medication category, discharge respiratory support, birth year, sex, and insurance type.

**Supplemental Table 6.** Association of community-level material deprivation (CMDI) with asthma diagnosis by age 5 years, restricted to infants without change in residential census tract in the first year after NICU discharge (n = 223).

|  | **OR** | **95% CI** | ***p*-value** |
| --- | --- | --- | --- |
| Unadjusted | 1.36 | 1.08-1.71 | 0.010 |
| Adjusted* | 1.38 | 1.02-1.87 | 0.034 |

*Model adjusted for gestational age, birth weight, BPD grade, discharge age (days), discharge inhaled medication category, discharge respiratory support, birth year, sex, and insurance type.
